# Outcomes of the NCI cancer prevention fellowship program in training multidisciplinary public health professional leaders

**DOI:** 10.1038/s41598-026-45502-4

**Published:** 2026-04-07

**Authors:** Shanen M. Sherrer, Jessica M. Faupel-Badger, Krista A. Zanetti, Heather R. Bowles, Katherine Dent, Tessa Swigart, Randy ZuWallack, Philip E. Castle

**Affiliations:** 1 Cancer Prevention Fellowship Program, National Cancer Institute, Bethesda, MD USA; 2 Department of Chemistry and Biochemistry, AAAS Science & Technology Policy Fellow, St. Mary’s College of Maryland, St. Mary’s City, MD USA; 3 Division of Research Capacity Building, National Institute of General Medical Sciences, Bethesda, MD USA; 4 Office of the Director, National Institutes of Health, Bethesda, MD USA; 5 ICF International, Rockville, MD USA

**Keywords:** Public Health postdoctoral training, Careers, Evaluation, Cancer prevention, Multidisciplinary training, Cancer prevention, Public health, Health occupations, Medical research, Preclinical research, Translational research, Oncology

## Abstract

Public health careers are varied, spanning different disciplines, employment sectors, and health concerns. In addition to those on the frontlines implementing public health programs, there is a need to develop researchers who integrate fundamental basic research, epidemiology, clinical, and social and behavioral sciences to create new interventions focused on specific diseases and improving health. Since 1987, the National Cancer Institute’s Cancer Prevention Fellowship Program (CPFP) has developed multidisciplinary leaders with expertise in cancer prevention who are collectively focused on decreasing cancer incidence and mortality. As part of this dual postdoctoral training in cancer prevention and public health research, CPFP has sponsored fellows without prior public health training to gain a Master of Public Health degree after earning their doctoral degree. In 2024, alumni (*n* = 189) and highly qualified applicants (*n* = 52) who did not join the program from 1987 to 2019 completed a survey to assess career outcomes. Survey responses demonstrated that CPFP alumni were employed across all career sectors (39.4% government, 39.5% academia, 10.8% private companies, and 10.3% other) and are retained in the research field (87.8%). Alumni credited the CPFP with being highly influential in their subsequent careers, demonstrating the valuable contribution of this multidisciplinary program in building the public health workforce.

**Supplementary Information:**

The online version contains supplementary material available at 10.1038/s41598-026-45502-4.

## Introduction

Public health education and training spans undergraduate, masters, predoctoral, and postdoctoral programs with impactful careers available at all training levels. Indeed, public health careers are varied with individuals trained in public health being employed across government, academic, non-profit and for-profit settings^1,2^. Among these careers are field epidemiologists, those leading public health programs for local, state, and national departments, and researchers who are developing the next generation of public health interventions. Public health professionals are prepared for these varied career paths because of the multidisciplinary nature of public health training. Individuals in formal public health education programs such as those earning Master of Public Health (MPH) degrees receive training in epidemiologic methods and biostatistics, the biological basis of disease, design and evaluation of public health interventions including social and behavioral approaches, and factors critical for success in implementing public health programs in a community^3,4^. Within the public health profession, there are individuals who focus on broadly implementing programs in collaboration with specialists who focus on a specific disease area such as cancer prevention and control^5^.

Cancer prevention and early detection efforts are critical to reducing United States cancer burden by decreasing cancer incidence and mortality^6,7^. Existing cancer prevention and early detection efforts have been cited as being responsible for 80% of the decrease in cancer mortality for breast, cervical, colorectal, lung, and prostate cancers combined from 1975 to 2020^6^. New opportunities for cancer prevention and early detection are rapidly increasing with the corresponding expansion of knowledge about the causes of cancer and the fundamental biological processes underpinning cancer. There is a need to have a robust, well-trained workforce poised to capitalize on these opportunities and develop new interventions and approaches for cancer prevention. This includes multidisciplinary cancer prevention researchers who can bring together these advances across disciplines and develop research programs aimed at ultimately decreasing cancer incidence and mortality. Key to achieving this goal is ensuring that these individuals have training in public health and can incorporate these tools and methods into their research. Notably, the National Cancer Institute Cancer Prevention Fellowship Program (CPFP) has been at the forefront of cancer prevention and control training since 1987.

CPFP is a postdoctoral program that recruits approximately 10 fellows a year on average who come from an array of disciplines (e.g., basic science, epidemiology/public health, clinical, and social and behavioral sciences) with less than 5 years of relevant postdoctoral experience at the time of the fellowship to focus their talents on cancer prevention through cohort-centered training and mentored research with available preceptors for up to 4 years. Since 1991, the program has also offered the opportunity for fellows without a background in public health to attain an MPH degree as part of their required postdoctoral training. The program now has approximately 400 alumni, with the vast majority receiving public health training while a fellow and incorporating this new knowledge into their careers. Here, we report on an evaluation of the career outcomes of CPFP alumni as the program approaches its 40th anniversary. This evaluation builds on a career outcomes evaluation conducted in 2014 which included CPFP alumni through 2011, thus there are over 100 additional alumni and 13 more years of outcomes since the last survey^8–11^. The results from this 2024 evaluation reinforce the value of the multidisciplinary training in CPFP, including the emphasis on gaining critical skills for public health research, in developing leaders in the field of cancer prevention and control, and in retaining individuals in the field.

## Results

### Respondents’ demographics

There were two populations of respondents for the survey – CPFP alumni who entered the postdoctoral program between 1987 and 2019 (noted hereafter as “alumni”) and applicants who were highly qualified and interviewed for a position but did not participate in CPFP (noted hereafter as “applicants”). While the most likely reason applicants did not participate was that they were not offered a fellowship, there were some applicants who chose not to participate in CPFP for various reasons. While there were no statistically significant differences in the demographic details of alumni and applicant respondent groups regarding age, sex, and race/ethnicity (Table 1), there were statistical differences in key professional details. For example, CPFP alumni completed their postdoctoral positions over a broad range of years (1987 to 2024) whereas applicant respondents mostly completed their postdoctoral positions between 2010 and 2024 (92.3%, *p* < 0.01). Markedly, 85.0% of alumni have an MPH with approximately 80% of these alumni earning their MPH through CPFP. It is presumed the remaining 15% of alumni had some other public health training (e.g., PhD in epidemiology) and did not need an MPH to complete their CPFP training.

**Table 1:** Demographic details of 2024 CPFP survey respondents.

	Alumni n (%) *N* = 189	Applicants n (%) *N* = 52	Fisher’s Exact Test
Age (years)*			
< 40	29 (18.8%)	12 (27.3%)	
40–49	64 (41.6%)	21 (47.7%)	
≥ 50	61 (39.6%)	11 (25.0%)	
Total	154 (100%)	44 (100%)	0.16
Sex			
Male	44 (25.6%)	10 (21.3%)	
Female	123 (71.5%)	35 (74.5%)	
Total	172 (100%)	47 (100%)	0.71
Race/Ethnicity			
White, non-Hispanic	115 (68.9%)	28 (60.9%)	
Other/Multiple	52 (31.1%)	18 (39.1%)	
Total	167 (100%)	46 (100%)	0.37
Cohort for Training Completion			
1987–2009	77 (41.4%)	***	
2010–2024	109 (58.6%)	36 (92.3%)	
Total	186 (100%)	39 (100%)	< 0.01
Degree			
PhD	161 (93.6%)	43 (91.5%)	
MPH	146 (85.0%)	19 (48.7%)	
Other	11 (6.40%)	***	
Total**	172 (100%)	47 (100%)	0.53
Discipline**			
Behavioral or social sciences	78 (43.0%)	21 (43.0%)	> 0.99
Biological or biomedical sciences	35 (19.0%)	***	0.84
Epidemiology and/or public health	121 (67.0%)	18 (37.0%)	< 0.01
Medicine	23 (13.0%)	***	0.49
Nutrition sciences	27 (15.0%)	***	0.15
Mathematical sciences, Physical sciences, or Other	35 (19.0%)	14 (29.0%)	0.17
Number of Disciplines			
One	86 (47.5%)	28 (57.1%)	
More than one	95 (52.5%)	21 (42.9%)	
Total	181 (100%)	49 (100%)	0.26
Employment Status			
Currently employed	185 (98.4%)	50 (96.2%)	
Unemployed/retired	***	***	
Total	188 (100%)	52 (100%)	0.30
Current Employer			
Government	73 (39.4%)	***	
NCI	35 (18.9%)	***	
National Health Institutes (NIH) other than NCI	18 (9.70%)	***	
Government agency other than NIH	20 (10.8%)	***	
University or some other academic institution	73 (39.5%)	27 (54.0%)	
Private company	20 (10.8%)	11 (22.0%)	
Other^#^	19 (10.3%)	***	
Total	185 (100%)	50 (100%)	< 0.01
Years in current job			
< 5	92 (50.3%)	33 (66.0%)	
5–9	34 (18.6%)	***	
≥ 10	57 (31.1%)	10 (20.0%)	
Total	183 (100%)	50 (100%)	0.15

*<40 = people born in 1985 and later; 40 – 49 = people born in 1984 − 1975; ≥ 50 = people born in 1974 and earlier.

**Category can equal more than 100% since respondents were allowed to select more than one answer in survey. The n displayed reflect total number of individual respondent alumni and applicant for this specific question.

***Due to *n* ≤ 10, data was suppressed to reduce risk of identity of survey respondents.

^#^Other includes independent cancer research center or some other health research institution, healthcare clinic or hospital, a foundation or professional association, and self-employed.

When describing current employment, CPFP alumni were more likely to work within the epidemiology and public health fields (*p* < 0.01) than the corresponding applicants. Alumni were also more likely to work in government whereas most applicants worked in academic or private sectors. Alumni were non-significantly more likely to work in multiple disciplines (52.5%) than applicants (42.9%, *p* = 0.26). Both alumni and applicants were in a wide range of current positions (Supplementary Fig. S1) across the government, academic, and other job sectors. In addition, many of the alumni (50.3%) and applicants (66%) had been in their current positions for 5 years or less, indicating most had experienced a recent career transition.

### Careers within the field of cancer prevention and control

**Fig. 1:**
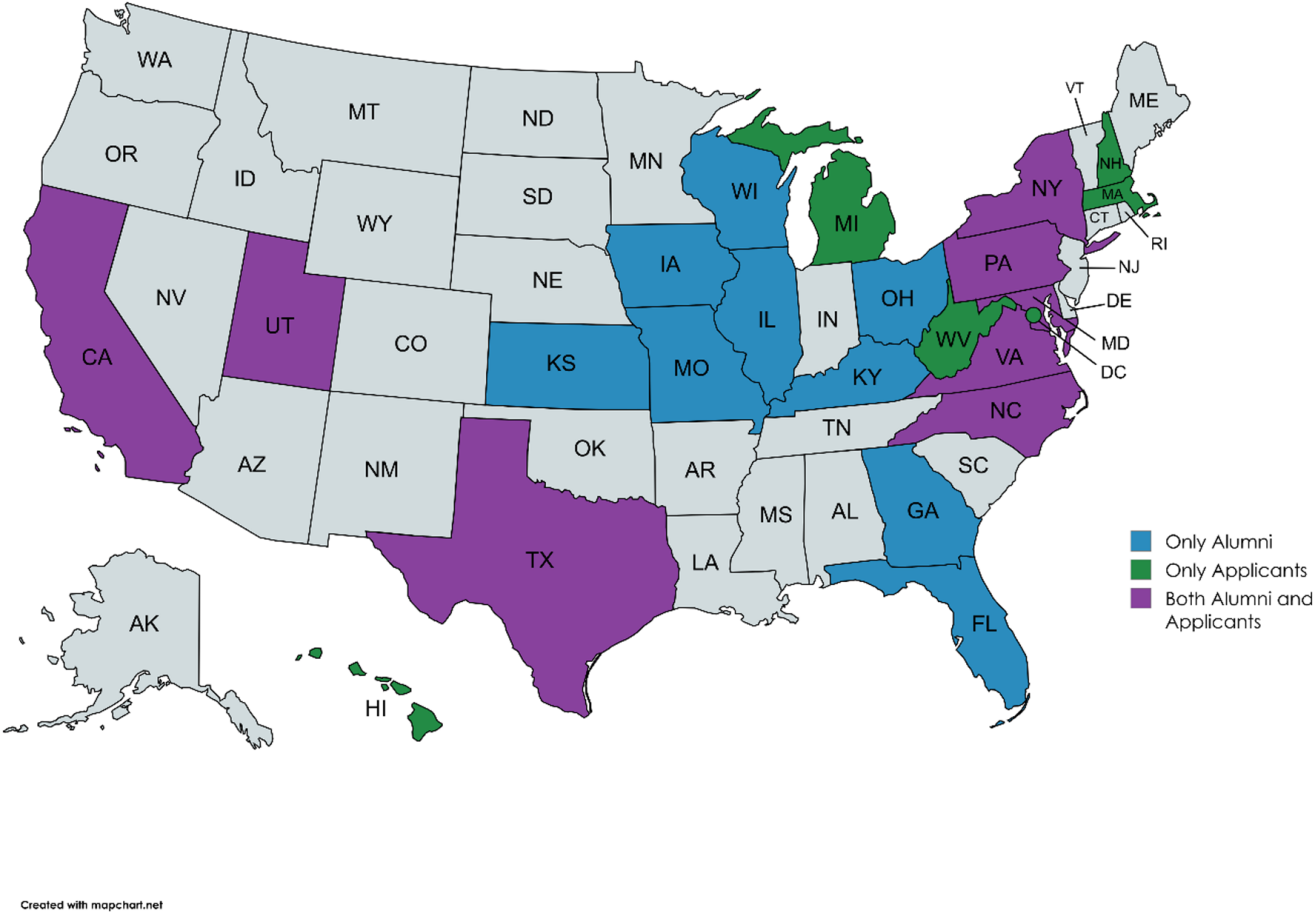
Affiliations with NCI-designated cancer centers of survey respondents. The CPFP alumni (blue colored states) and applicants (green colored states) of cancer center affiliations are shown to visualize distribution of work.

Affiliation with an NCI-designated Cancer Center was analyzed since cancer prevention and control work is a multidisciplinary field that includes translational research, and these centers are leading institutions for cancer prevention and control programs (Fig. 1). Among individuals in academic positions 38.6% of applicants (*n* = 17) and 35.1% of alumni (*n* = 39) reported being affiliated with an NCI-designated Cancer Center (Table 2). Of those affiliated with cancer centers, 34.2% of alumni (13.2% of all alumni) and 11.8% of applicants were in leadership positions (*p* = 0.11). Overall, regardless of employment sector, a majority (87.9%) of CPFP alumni reported working within the cancer prevention and control field (Table 3), and this was significantly greater than applicants reporting working in the field (59.2%, *p* < 0.01). Alumni also reported that their work involved conducting research in the field (90.4%), compared to 71.5% of applicants (*p* < 0.01, Table 3). Among the 12.2% of alumni (*n* = 22) and 40.8% of applicants (*n* = 20) not currently working in cancer prevention and control, their reasons were statistically similar, including but not limited to finding a better opportunity outside of the field, career/professional interests changing, and not having a suitable job in the field^12^.

**Table 2:** Affiliations and roles within NCI-designated cancer centers.

	Alumni *N* = 111 (%)	Applicants *N* = 44 (%)	*p* value^a^
Affiliated with an NCI Cancer Center			
Yes	39 (35.1%)	17 (38.6%)	
No	72 (64.9%)	27 (61.4%)	0.71
Role at NCI Cancer Centers			
Member	26 (65.8%)	15 (88.2%)	
Leadership Role	13 (34.2%)	* (11.8%)	0.11

^a^Calculated using Fisher’s Exact Test.

*Due to *n* ≤ 10, data was suppressed to reduce risk of identity of survey respondents.

**Table 3:** Reported work within cancer prevention and control field for both CPFP alumni and applicants.

	Alumni n (%) *N* = 181	Applicants n (%) *N* = 49	Fisher’s Exact Test
Any Cancer Prevention and Control Work^a^			
None (0%)	22 (12.2%)	20 (40.8%)	
Some (1–50%)	70 (38.7%)	14 (28.6%)	
Majority (≥ 50%)	89 (49.2%)	15 (30.6%)	
Total	181 (100%)	49 (100%)	< 0.01
Cancer Prevention and Control Research^b^			
None (0%)	17 (9.60%)	12 (28.6%)	
Some (1–50%)	78 (44.1%)	17 (40.5%)	
Majority (≥ 50%)	82 (46.3%)	13 (31.0%)	
Total	177 (100%)	42 (100%)	< 0.01

^a^Work excludes time spent on cancer treatment or cancer treatment research.

^b^Time of work includes conducting research themselves as well as time spent supporting the research of others through activities such as research management, monitoring, reviewing, funding, analysis, dissemination, and mentoring.

When mapping general career trajectories as a function of CPFP experiences, a multiple logistic regression model was constructed, and several associations were identified (Table 4). For example, both being a CPFP alumni as well as training in epidemiology were highly correlated with cancer prevention and control work and research. When compared to the applicants without epidemiology training, applicants with epidemiology training (odds ratio (OR) = 11.0, 95% confidence interval (CI): 1.7–69.1), alumni with epidemiology training (OR = 9.5, 95% CI: 2.0–45.0), and alumni without epidemiology training (OR = 6.0, 95% CI: 1.2–30.2) were more likely to work in cancer prevention and control. Similarly, when compared to the applicants without epidemiology training, alumni with epidemiology training (OR = 10.6, 95% CI: 2.1–54.2) were more likely to do cancer prevention and control research as were alumni without epidemiology training and applicants with epidemiology albeit not statistically significant. Oddly, working within the biomedicine discipline was negatively correlated with doing cancer prevention research (OR = 0.36, 95% CI: 0.15–0.86). Note that in the 2024 survey the biological/biomedical sciences as a scientific discipline option represented more laboratory-based research – spanning basic science to clinical and translational work. Additionally, while more than one scientific discipline could be selected, respondents with epidemiology and/or public health were more likely to be in the field of cancer prevention and control independent of other scientific discipline variables. There were no statistical differences in predicting work within the field for scholars and researchers at early career (cohorts 2016 to 2024), mid-career (cohorts 2009 to 2015) or late career (cohorts 2008 and earlier years) stages. Age, sex, and race/ethnicity were not predictive for a cancer prevention and control career and therefore these variables were not added to the model described above (Table 4).

**Table 4:** Odds ratios for factors associated cancer prevention and control work and research.

	Cancer Prevention and Control Work Odds Ratio (95% CI)			Cancer Prevention and Control Research Odds Ratio (95% CI)	
Cohort	*n*			*n*	
Late career	34	1.0 (referent)		33	1.0 (referent)
Mid career	84	0.5 (0.2–1.2)		80	0. 6 (0.2–1.4)
Early career	98	0.4 (0.2–1.1)		94	0.76 (0.3–2.1)
Doctoral Degree					
PhD	191	1.0 (referent)		185	1.0 (referent)
Other	25	0.7 (0.3–1.9)		22	1.1 (0.4–3.2)
Social/Behavioral Science					
No	126	1.0 (referent)		119	1.0 (referent)
Yes	90	1.7 (0.9–3.2)		88	1.9 (1.0-3.5)
Biomedicine					
No	175	1.0 (referent)		168	1.0 (referent)
Yes	41	0.4 (0.2–0.9)		39	0.4 (0.2–0.9)
Employer					
Other	40	1.0 (referent)		37	1.0 (referent)
Government	100	1.4 (0.6–3.5)		97	1.4 (0.5–3.6)
University/research center	76	2.9 (1.2-7.0)		73	3.3 (1.3–8.3)
Years in Current Job					
< 5	113	1.0 (referent)		107	1.0 (referent)
5–9	41	0.6 (0.3–1.4)		40	0.6 (0.3–1.5)
≥ 10	62	0.7 (0.3–1.6)		60	0.7 (0.3–1.6)
Alumni or Applicants in Epidemiology					
Applicants without Epidemiology	21	1.0 (referent)		15	1.0 (referent)
Applicants with Epidemiology	16	11 (1.7–69.1)		16	5.3 (0.8–34.5)
Alumni with Epidemiology	121	9.4 (2.0–45.0)		119	10.6 (2.1–54.2)
Alumni without Epidemiology	58	6.0 (1.2–30.2)		57	3.1 (0.6–16.7)

Multiple logistic regression was used to calculate the adjusted odds ratios between the dependent variables and the two study populations (alumni and applicants) after adjusting for the other covariates in the model.

Notably, CPFP alumni were more likely to report being extremely satisfied with their career (24.9%) than applicants (4.3%) (*p* < 0.01). With career satisfaction being often linked to career promotions, professional advancements such as being in leadership positions were also examined. Survey respondents were more likely to be in a leadership position when in the academic sector (48.3%) than government (27.3%) or other (24.5%) job sectors (*p* = 0.01). Overall, there was no significant difference between CPFP alumni (63.7%) and applicants (55.1%) being in a leadership position at the time of the 2024 survey (*p* = 0.18). When examining other types of career advancements, only the employment activities and accomplishments of CPFP alumni were analyzed due to small sample sizes.

Regardless of position, CPFP alumni frequently performed tasks related to multidisciplinary activities and scientific contributions to cancer research (Supplementary Fig. S2). These activities have led to a wide range of professional recognition for their work. As an example accomplishment, one person reported “*establishing a new area of cancer research […]*,* [and] successful transition to different work environments (government*,* non-profit*,* etc.).*” Another respondent accomplished “*creating a qualitative program involving focus group analyses to better understand perceptions related to cancer prevention.*” Collectively, alumni shared accomplishments (Table 5) themed around scientific contributions and innovation, career advancement and recognition, and public health and community impact. These themes were evident in another participant’s self-reported accomplishments:

**Table 5:** Reported career accomplishment summaries of 2024 CPFP survey alumni respondents.

Themes	Alumni
Scientific Contributions and Innovation	Contributing to scientific knowledge and advancing research (*n* = 70). Publishing high-quality research and influential papers in high-impact journals (*n* = 26). Leading clinical trials and developing new intervention strategies (*n* = 9). Conducting clinical trials and significant research studies (*n* = 18). Developing new research programs and initiatives (*n* = 28).
Career Advancement and Recognition	Securing tenure and senior leadership roles (*n* = 21). Achieving rapid career progression and prominent positions (*n* = 21). Balancing professional achievements with personal responsibilities (e.g., parenting) (*n* = 3). Holding leadership positions within organizations and institutions (*n* = 20). Being appointed to prestigious boards and committees (*n* = 5). Receiving honors and awards for contributions to the field (*n* = 10).
Public Health and Community Impact	Contributing to public health initiatives and policy advocacy (*n* = 8). Developing community programs and outreach efforts (*n* = 6). Establishing guidelines and policies for health interventions (*n* = 17).

Because this was an open-ended question on the survey, the responses were coded into overarching award type categories presented in the table above. Note that the themes from the optional responses may have come from more than one respondent, and/or one respondent having a combination of themes.

*“The ability to make programmatic decisions and promote areas of research for funding. Understanding priorities for scientific research in my area of expertise and align those with NIH [institutes/centers/offices] goals and objectives. Leading the development of strategic plan for nutrition research in the area of microbiome and its relationship in health and disease […].”*

### Reflections on CPFP experiences

**Fig. 2:**
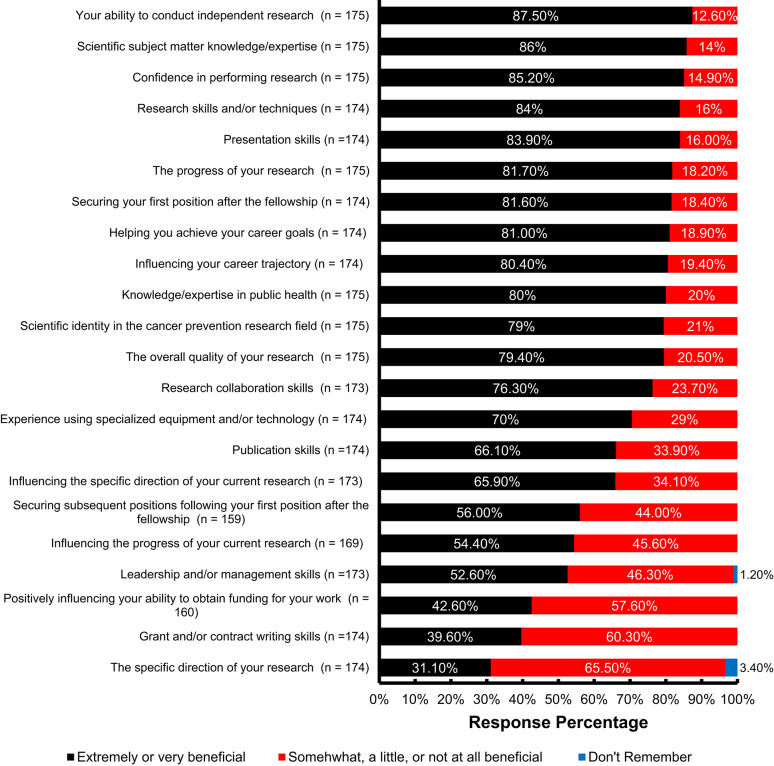
Beneficial aspects of CPFP according to alumni’s career outcomes. CPFP alumni indicated specific aspects of the program (vertical items) that were somewhat, a little, or not at all beneficial (red bars); extremely or very beneficial (black bars); or don’t remember (blue bars).

CPFP alumni named multiple program aspects that laid the foundation of their professional successes (Fig. 2). At least 70% of alumni said that CPFP was extremely or very beneficial to their knowledge, skills, and most research management areas except for specific direction of their research. Additionally, at least 50% of alumni said that the fellowship was extremely or very beneficial to their publication, presentation, and leadership and/or management skills. In terms of career progressions, over 80% of alumni reported that CPFP was extremely or very beneficial to helping them secure their first position after the fellowship as well as helping them achieve their career goals and influencing their career trajectory.

When highlighting the most valued aspect of CPFP (Table 6), alumni cited academic and professional growth component of the program (41.1%) the most. From their perspective, this component included the opportunity to earn an MPH, access to mentored research training, and building/refining leadership and presentation skills. For example, one person said that the most valuable aspect of CPFP was “*the ability to receive an MPH degree at one of the top ranked public health schools in the world and then to apply that knowledge in combination with past laboratory skills gained during my graduate studies*.” Others stated that CPFP provided “*a wider view of prevention and Public Health*”, “*the cohort experience*” of postdoctoral “*interdisciplinary training*”, and “*opportunity to work on large multi-disciplinary projects.*” Most important, 98% of CPFP alumni respondents said that they would participate in the program again, and 97% of the CPFP alumni said that they would recommend others to the program.

**Table 6:** Most valued parts of CPFP cited by alumni.

Themes	Theme Summaries
Academic and Professional Growth (*n* = 46)	MPH Degree: Multiple fellows highlighted the value of obtaining an MPH degree, which opened new career opportunities and provided a broad perspective on public health (*n* = 26). Research Training: The fellowship provided extensive research training, enabling fellows to pivot to new fields and gain hands-on epidemiological and statistical skills (*n* = 10). Leadership and Presentation Skills: Structured training in leadership and effective communication was critical for professional development (*n* = 10).
Multidisciplinary and Collaborative Environment (*n* = 28)	Multidisciplinary Training: Exposure to a wide range of disciplines and the opportunity to work in multidisciplinary teams broadened fellows’ perspectives and skills (*n* = 18). Collaborative Culture: The collegial environment fostered collaboration and camaraderie among fellows from different backgrounds (*n* = 10).
Program Structure and Support (*n* = 25)	Structured Program: The well-organized program, including mandatory trainings and a cohort model, provided a strong foundation for professional and personal growth (*n* = 16). Funding and Resources: Financial support for research and access to resources at NCI and NIH were crucial for conducting high-quality research (*n* = 9).
Personal Development and Opportunities (*n* = 13)	Career Transition: The fellowship provided the time and support needed to transition to new fields or research areas, helping fellows to redefine their career paths (*n* = 3). Personal Growth: The experience boosted fellows’ confidence and provided opportunities for independent learning and exploration (*n* = 10).

Because this was an open response option, responses were categorized into the above themes and quantified the number of responses that related to each theme. Note that the themes from the optional responses may have come from more than one respondent, and/or one respondent having a combination of themes.

## Discussion

The field of cancer prevention and control is focused on the public health goals of decreasing cancer incidence, morbidity, and mortality. Since cancer is not a single disease, there are complexities in cancer prevention and control – variability in causation and onset of disease, challenges in identifying intervention points, human behavior, and evolving understanding of survivorship as treatments improve. As such, the field of cancer prevention and control is a specific and rapidly evolving public health field. All these complexities and considerations are impetus for training scientists to be multidisciplinary.

Individuals working across the translational spectrum and disciplines such as epidemiology, biology, clinical, social, and behavioral science are needed to develop and implement interventions that will enable the cancer prevention and control field to achieve these public health goals. In this field, potential leaders are individuals who are not only experts in their area(s) but are conversant across the multiple disciplines needed to develop effective cancer prevention and control interventions, as well as the levels of evidence and public-health approaches needed to bring a research advancement to the population level. Since 1987, CPFP has been at the forefront of training leaders in this field. In addition to CPFP being at the forefront of scientific research, it has also been a model of multidisciplinary, comprehensive postdoctoral training. This training model includes experiences such as leading interdisciplinary collaborations, professional skill development, leadership training, and creating a sense of a professional community^3,13–18^. The value of such postdoctoral training programs can be measured via evaluation that includes the aforementioned components as well as gaining input from all members of the program community (i.e., advisors and trainees), monitoring differences between trainees’ career trajectory and initial professional goals, and tracking career transitions post-training^4,13,19–21^. The work presented herein reflects this practice in a public health field – cancer prevention and control. In 2014, the CPFP completed its first comprehensive outcomes evaluation and established the baseline performance of the postdoctoral program in the context of alumni perspectives, alumni successes, and programmatic impacts within the cancer prevention and control field^8–11^. This evaluation of CPFP that included alumni and control group perspectives was able to capture these considerations while highlighting career outcomes from multidisciplinary postdoctoral training in cancer prevention and control at the population level.

Based on the 2024 CPFP survey responses, individuals who either completed the CPFP (alumni) or applied but did not ultimately join the CPFP (applicants) had career outcomes that spanned across all job sectors and career stages (Table 1 and Supplementary Fig. S2). While programs supporting researchers becoming multidisciplinary or gaining additional formal education have been noted in the past,^15,17,22^ many training programs prepare participants for a limited range of careers^15^. CPFP participation markedly increased the probability of being active within the multidisciplinary cancer prevention and control field (from 32.5% in 2014 to 52% in 2024) as evident by the positive correlation between being a CPFP alumni with epidemiology training (60% or 1.6-fold increase from 2014 evaluation^8^. and conducting cancer prevention and control research (Table 4, a slight decrease of 1.1- to 1.2-fold from 2014 evaluation^8^. The negative association between being a biomedical scientist and doing either work or research in the field, which is a 1.6- to 3.0-fold decrease in correlation from the predictive model based on the 2014 evaluation^8^, was most likely due to the varying definition of biomedical sciences and traditional medicine as scientific research fields^23–26^. In addition, the career trajectories of CPFP alumni (Table 5) were supported by hallmark components of the program – professional/personal growth, leadership training, multidisciplinary and collaborative environments, research support, cohort-based training, and providing public health skills relevant to cancer prevention. Thus, these CPFP career and professional development components ensure training of transferrable skills across multiple career sectors, which is central to postdoctoral training in general and highly relevant to jobs with a focus on significantly decreasing cancer mortality and incidence^5–7,27,28^.

The programmatic success of the CPFP is consistent with expectations for a researcher being part of a postdoctoral training program to increase their publication impact and level of engagement within specialized fields of study as well as promote interdisciplinary research when compared with postdoctoral researchers not participating in a formal training program^3,15,18^. For example, CPFP fosters a multidisciplinary research approach that ensures all fellows have the training to understand cancer prevention as a public health issue – a viewpoint advantageous for future leaders of the field. Additionally, it was observed that the level of research activities within cancer prevention and control field (90.4%) and job relevance (87.9%) for CPFP alumni respondents of the 2024 survey (Table 3) are higher than other biological and life science PhD holders in either academia or private sector (62.7% and 76.5%, respectively) of a previous study^28^. To promote the advancement of multidisciplinary cancer research, the CPFP components are geared towards future research productivity with postdoctoral scholars refining skills such as project management and leadership^27^. Notably, the frequent career activities of alumni (Supplementary Fig. S2) can be directly mapped to the knowledge, skills, and research experiences benefited from CPFP participation (Fig. 2). Furthermore, these benefits can be aligned to the valued CPFP foci (Table 6). Many of the alumni valued the opportunity to earn an MPH degree, cohort experiences, multidisciplinary research exposure, and leadership training (Fig. 2). Skills gained through these experiences are beneficial for leadership/management positions supporting the development of the next-generation of scientists within the cancer prevention and control field (examples in Table 2 and Supplementary Fig. S2), which are crucial to direct the advancement of the scientific field as well as identify what infrastructure is needed to sustain the research momentum^27,29^. Seeking and filling these high-impact roles should be considered activities that reflect career satisfaction with measurement achievements along their professional trajectory^21,30^. In addition, these activities along with career satisfaction directly can measure the significance and impact of CPFP on alumni’s career outcomes within a public health related field.

In evaluation of postdoctoral training programs, inclusion of comparison groups or cohorts is often omitted since using appropriate comparison groups for postdoctoral training program evaluations is complicated^13^. The 2014 CPFP evaluation^8–11,22^ included applicants who were eligible for but were not enrolled into the program as the control group, which optimally highlighted specific programmatic aspects of career outcomes. Similarly, a 2017 programmatic evaluation of the Fellowships in Research and Science Teaching (FIRST, established in 2000) postdoctoral program at Emory University had two comparison groups for those who did not participate in FIRST – specific T32 fellows at the university and other institutional research and academic career development award institutions, and sometimes national data for T32 grants.^[14 8–11,22^. The approach to include a comparision group, though small, was continued for the 2024 CPFP evaluation, it was in 2014, when appropriate to link relevant outcomes to programmatic training aspects for the workforce within the field of cancer prevention and control.

A notable limitation of the 2024 CPFP survey is that individuals who responded were likely to be the ones with more positive career outcomes, which extended to the low survey response rate for applicant group. It would be highly unlikely an applicant with no connection to CPFP would have responded if the person did not have perceived strong career outcomes to report and/or did not want to recall requested information, as observed in other evaluation studies^31,32^. While this is also a caveat for CPFP alumni respondents, there was a response rate of over 70% among the program alumni. Thus, this response bias existed for both population groups of the survey, with possible attenuation for alumni due to the strong response rate.

In conclusion, the 2024 CPFP evaluation showed participants’ career outcomes after this postdoctoral program reflect the program’s goals – providing comprehensive yet flexible postdoctoral training that supports mentored multidisciplinary research opportunities and leads to successful careers within the public health field of cancer prevention and control. This evaluation also provided evidence that the program alumni contributed positively to the field. In addition, the 2024 CPFP survey data highlighted program aspects most impactful for alumni and multidisciplinary research within this field with public health implications. These data are important not only for understanding alignment of training with career trajectories in the field of cancer prevention but also demonstrate the value of formally incorporating public health training into postdoctoral programs aligned with health goals at the population level. Furthermore, this evaluation provides more data to understand outcomes of cohort-based postdoctoral training and postdoctoral training in general.

## Methods

### Study populations

The NCI CPFP, headquartered in Rockville, Maryland, USA, provides a rigorous, selective postdoctoral program that is focused on cancer prevention. CPFP interviews about 20 applicants for each cohort annually, for which they accept about 10–15 fellows. The NCI CPFP fellowship has been described in detail elsewhere^1–3^. For this evaluation, as with a preceding study conducted in 2012^4^, the population had two types of respondents, referred to in this paper as “alumni” and “applicants.” The main population of interest included any alumni of the CPFP program since it first started in 1987 who had participated as an NCI Cancer Prevention Fellow for at least 12 months. The survey was sent to alumni who entered the program as of August 31, 1987, through to those who entered the program no later than December 31, 2019. Of the 355 people who met this inclusion criteria, 32 were excluded (8 were deceased, and 24 had no active contact information we could locate). After sending the initial recruitment email and survey link, there were 62 bounce-back emails. This left a total alumni sampling frame of 261. A summary of participation from eligible survey respondents can be found in Supplementary Table S1.

The study also included a comparison population; all applicants to the CPFP in the same timeframe as above as those who were invited to interview for the fellowship by October 2019 but were ultimately not enrolled into the program. Applicants who were interviewed but not selected — as opposed to the entire applicant pool — were considered as representative as possible to alumni for comparison because of their equivalent education level and shared interest in cancer prevention. There were 569 eligible applicants, of which 96 were excluded because they were deceased or had no contact information we could locate. After the initial recruitment email was sent, there were 153 bounce-back emails, leaving a total applicant sampling frame of 320 (Supplementary Table S1). The information collected at the time of CPFP application submissions for non-responding applicants was not included in the data analysis since this information was not up to date and could not be compared to more recent career outcomes of applicant respondents.

### Instrument development and survey implementation

This program evaluation was a 13-year follow-up of a prior one conducted in 2014^4^. The development of the original survey instrument is described in detail elsewhere^4^. Briefly, the questions in the survey were based on evidence from a literature review of prior studies looking at training program evaluations, as well as insights from in-depth interviews with CPFP alumni. The 2024 survey instrument was nearly identical to the survey developed in 2011, except for a few additional questions and prompts relating to work history and achievements, how many years they had in postdoctoral training, the title of their current position, their affiliation with an NCI-designated Cancer Center, and updated demographic questions to reflect recent best practices for collecting those data. The 2024 survey can be seen in Supplemental Fig. S3, which included how informed consent from human participants were obtained.

The survey instrument, along with the recruitment email, was originally sent from the survey platform, Qualtrics. Sending the email and link through Qualtrics allowed the contractor team (ICF) to accurately and efficiently track bounce-back emails as well as the timing of respondents who opted out of future emails and those who opened and/or completed the survey. Institutional review board (IRB) of ICF approved this study and gave this study exempt status (project #2024-066). All data collection and analysis procedures were compliant with the ethical standards outlined in ICF’s IRB, and all data were kept on secure, password protected servers and only accessible to ICF study personnel. The survey launched on May 14, 2024, and closed on July 17, 2024. During the active data collection period, ICF sent reminder emails from Qualtrics three times. To encourage participation, the director of the CPFP program sent recruitment emails with the survey link three additional times from her NCI email account.

### Outcome measures

The main objective of this study was to understand the relationship between participation in CPFP and working in cancer prevention and cancer prevention research. Therefore, the main outcome indicator in the survey was the question — which asked them to exclude cancer treatment and cancer treatment research — “Approximately what percentage of your current work is done in cancer prevention and control?” The percentage amounts they could choose from included none (0%), a small percentage (1%-25%), a moderate percentage (26%-50%), a large percentage (51%-75%), or a very large percentage (76%-100%). The follow-up questions were arranged based on the initial response to this question, including overall research and research support activities and, if indicated by the respondent, reasons why they were not working within the public health related field of cancer prevention and control.

### Statistical analysis

A bivariate analysis was performed to analyze crude differences between alumni and applicants using Fisher’s exact test. To depict participant demographic information and other characteristics, the variables were tabulated and corresponding distributions calculated for age (< 40, 40–49, or ≥ 50), sex (male or non-male), race/ethnicity (white, non-Hispanic, or other than white), training completion cohort (by decade years), cohort [defined as early career (those who finished their postdoctoral training between 2014 and 2024), mid-career (those who finished their postdoctoral training between 2004 and 2013) and late career (those who finished their postdoctoral training in 2003 or prior)], doctoral degree (PhD or other), career discipline and number of disciplines (behavioral or social sciences, biological or biomedical sciences, epidemiology and/or public health, medicine, nutrition sciences, mathematical sciences, or other), employer type (government, academic, or other), and years at current job (< 5, 5–9, ≥ 10).

Logistic regression was conducted to investigate the associations between the dependent variables — time spent on cancer prevention and control and conducting cancer prevention and control research — and the two study populations, alumni and applicants. Dependent variables were examined dichotomously by analyzing responses as ≥ 50% or < 50% time spent on cancer prevention or research. A logistic regression model was run with all variables mentioned above excluding age, sex, race/ethnicity, and training completion cohort variables. These variables were evaluated for inclusion in the model but ultimately eliminated since they were not significant predictors of cancer prevention research, and they contained missing responses that were reducing the number of valid responses for estimating the model.

## Supplementary Information

Below is the link to the electronic supplementary material.

Supplementary Material 1

## Data Availability

The aggregate data that support the findings of this study are available in the Methods, Results and Supplementary Information sections of this article. Other data that are not shown in this article are available on reasonable request from the corresponding author. The raw survey data are not publicly available due to privacy concerns and restrictions (ICF IRB exempt #2024-066).
