# Supplementary material for: Outcomes of the NCI cancer prevention fellowship program in training multidisciplinary public health professional leaders
Source: Sci Rep. 2026 Apr 7;16:16594. doi: 10.1038/s41598-026-45502-4 (PMC13219630; doi:10.1038/s41598-026-45502-4)
Supplement: Supplementary file 1 — Supplementary Material 1 [file 41598_2026_45502_MOESM1_ESM.pdf]

Supplementary Information:

Outcomes of the NCI Cancer Prevention Fellowship Program in Training Multidisciplinary Public Health Professional Leaders

Shanen M. Sherrer[1,2,+], Jessica M. Faupel-Badger[3,+], Krista A. Zanetti[4], Heather R. Bowles[1], Katherine Dent[5], Tessa Swigart[5], Randy ZuWallack[5], Philip E. Castle[1*]

Affiliations Noted:

[1]Cancer Prevention Fellowship Program, National Cancer Institute, Bethesda, MD

[2]AAAS Science & Technology Policy Fellow, Department of Chemistry and Biochemistry, St. Mary's College of Maryland, St. Mary's City, MD

[3]Division of Research Capacity Building, National Institute of General Medical Sciences, Bethesda, MD

[4]Office of the Director, National Institutes of Health, Bethesda, MD

[5]ICF International, Rockville, MD

[+]Shanen M. Sherrer and Jessica M. Faupel-Badger are co-first authors.

*Corresponding author: philip.castle@nih.gov

Fig. S1.

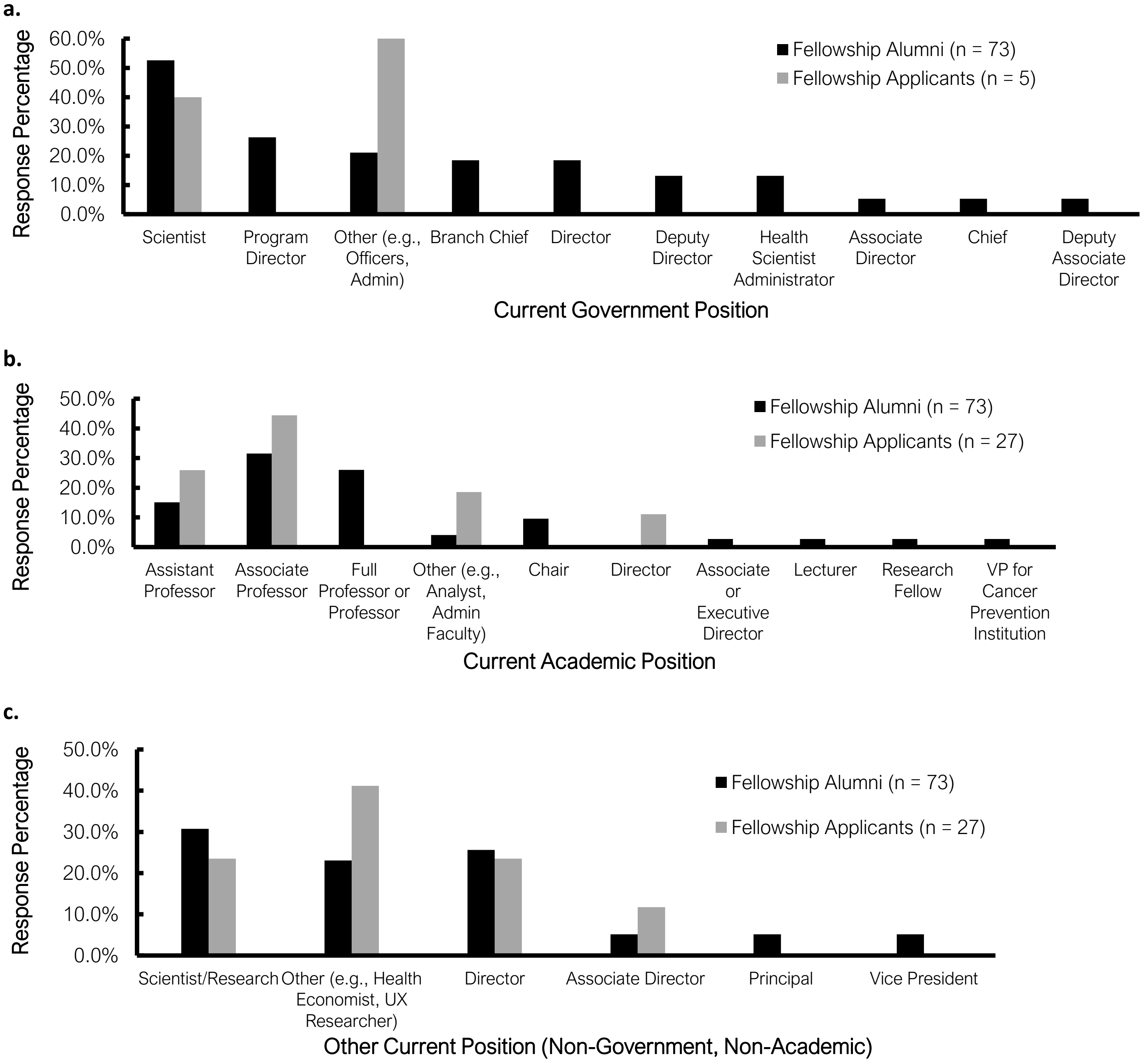

Supplementary Fig. S1. CPFP alumni and applicant current positions categorized by job sectors. The reported percentage of alumni (black bars) and applicants (grey bars) employment position categories are shown for (**a**) government, (**b**) academic, and (**c**) other job sectors. For each sector, the population numbers for alumni and applicants are shown within the figure legend of the images. The position category "Other" within the government job sector included reported positions not shown within the government sector. The position category "Other" within the academic job sector included reported positions not shown within the academic sector. The position category "Other" within the other job sectors included reported positions not shown within non-academic and non-government job sectors. Please note that the "Other" job sector includes all sectors that did not overlap with government and/or academic job sectors.

Fig. S2.

a.

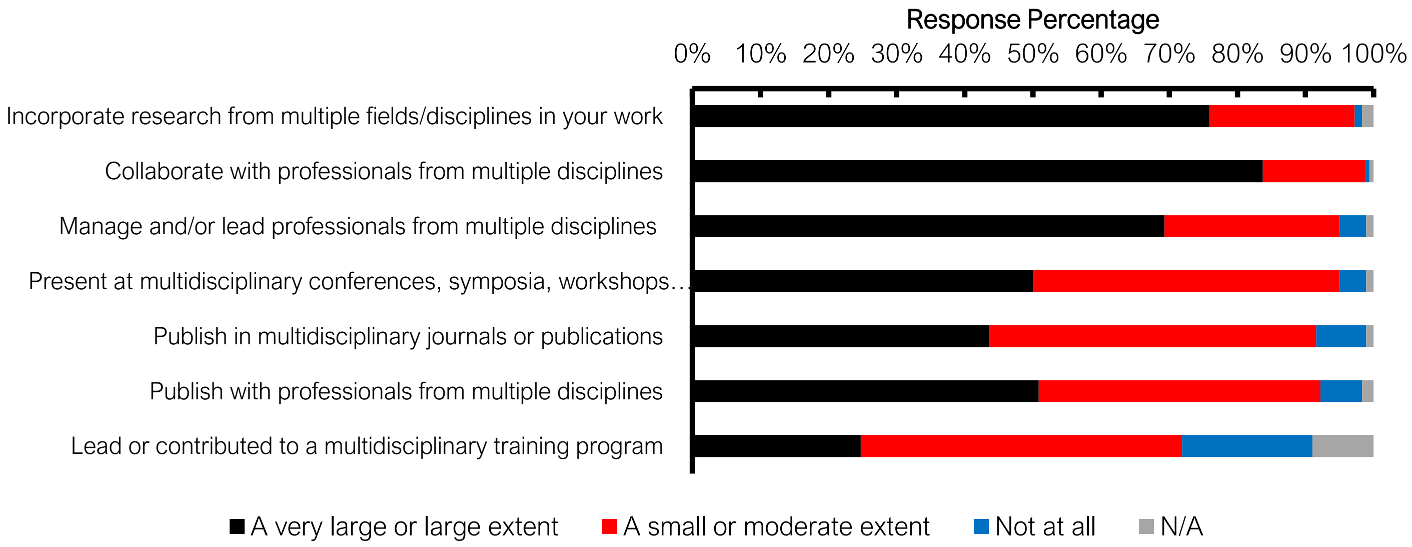

b.

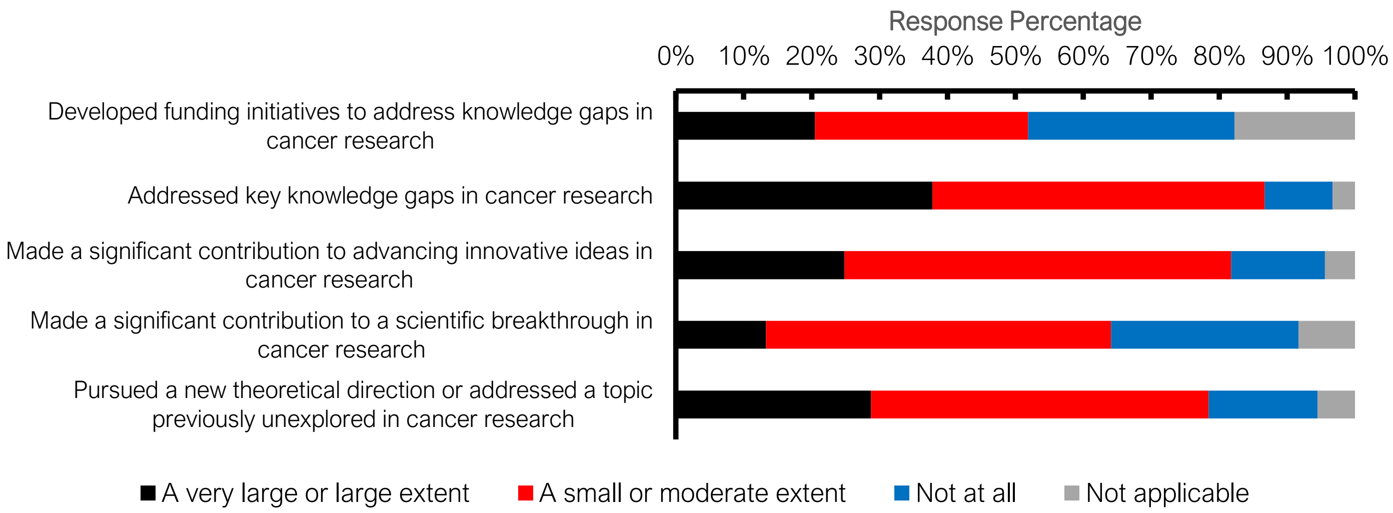

Supplementary Fig. S2. Current employment activities of CPFP alumni. Within the alumni's current positions, related (**a**) multidisciplinary activities and (**b**) scientific contributions were rated in frequency of specific tasks (vertical items) as small or moderate extent (red bars); a very large or large extent (black bars); not at all (blue bars); or not applicable (grey bars).

Table S1.

**Table S1.** Inclusion and exclusion criteria in survey participation for CPFP alumni and applicants.		
	**Alumni**	**Applicants**
Total number of eligible respondents	355	569
Total number excluded from recruitment outreach due to being deceased or could not locate contact information	32	96
Bounce-back emails after initial recruitment	62	153
Total sampling frame	261	320
Total complete responses (response rate)	189 (72%)	52 (16%)

Fig. S3.

Supplementary Fig. S3. CPFP Survey instrument used in the 2024 study. The survey instrument (shown below) was administered by ICF and used to collect responses from CPFP alumni and applicants. Design and implementation details can be found within the main text Methods section.

CPFP Survey of Alumni and Applicants Instrument

Evaluation of the National Cancer Institute's (NCI) Cancer Prevention Fellowship Program (CPFP) Survey of Alumni and Applicants

EVALUATION OF THE NATIONAL CANCER INSTITUTE'S CANCER PREVENTION FELLOWSHIP PROGRAM SURVEY OF ALUMNI AND APPLICANTS

OMB No: 0925-0778
Expiration Date: 09/30/2026

Collection of this information is authorized by The Public Health Service Act, Section 411 (42 USC 285a). Rights of study participants are protected by The Privacy Act of 1974. Participation is voluntary, and there are no penalties for not participating or withdrawing from the study at any time. Refusal to participate will not affect your benefits in any way. The information collected in this study will be kept private to the extent provided by law. Names and other identifiers will not appear in any report of the study. Information provided will be combined for all study participants and reported as summaries. You are being contacted by email to complete this instrument so that we can evaluate the Cancer Prevention Fellowship Program.

Public reporting burden for this collection of information is estimated to average 25 minutes per response, including the time for reviewing instructions, searching existing data sources, gathering and maintaining the data needed, and completing and reviewing the collection of information. An agency may not conduct or sponsor, and a person is not required to respond to, a collection of information unless it displays a currently valid OMB control number. Send comments regarding this burden estimate or any other aspect of this collection of information, including suggestions for reducing this burden, to NIH, Project Clearance Branch, 6705 Rockledge Drive, MSC 7974, Bethesda, MD 20892-7974, ATTN: PRA (0925-0778). Do not return the completed form to this address.

The National Cancer Institute (NCI) has contracted with ICF, an independent research firm, to conduct a comprehensive evaluation of the NCI's Cancer Prevention Fellowship Program (CPFP). The purpose of the evaluation is to collect information on the careers and experiences of former fellows and people who applied to the CPFP. Your participation will help provide valuable information that will assist CPFP in making decisions about future program initiatives to improve postdoctoral training.

We anticipate the survey will take approximately 25 minutes to complete.

All information collected in this survey will be stored in a password-protected computer folder. Survey findings will be reported in aggregate and will not connect individuals or their organizations with their answers. All questions are optional, and you may exit this survey at any time. If you leave the survey early, you can re-enter later to complete it. If you have any questions about this research study, please contact the research manager, Tessa Swigart at Tessa.Swigart@icfnext.com. If you have any questions about your rights as a research participant, please contact the ICF IRB chair, Christine Walrath, at Christine.Walrath@icf.com.

By continuing, you indicate your consent to participate in the survey.

1. Which statement represents your experience with the National Cancer Institute (NCI) Cancer Prevention Fellowship Program (CPFP)? *(Please select one answer.)*

Previously applied to the CPFP and was interviewed but did not participate in the program	☐[1] *(Applicant group)*
Participated in the CPFP program..................	☐[2] *(Alumni group)*

EMPLOYMENT INFORMATION

The first section of the survey asks questions about your work history and the type of work you are currently doing.

2. For the following question, please include your participation in the Cancer Prevention Fellowship Program if applicable. Which statement represents your postdoctoral or fellowship experience? *(Please select one answer.)*

Completed **one** postdoctoral or fellowship program or position	☐[1]
Completed **multiple** postdoctoral or fellowship programs or positions	☐[2]
Have not completed a postdoctoral or fellowship program or position...	☐[3] *(Go to question 6)*

3. How many years in total were you in postdoctoral training position(s)?_______________________

4. In what year did you complete your most recent postdoctoral or fellowship position? [Drop down with years 1980-2024]

5. Do you **currently** hold a postdoctoral or fellowship position?

Yes	☐[1]
No..	☐[2]

6. Including self-employment, what is your **current** employment status? *(Please select one answer.)*

Employed full-time	☐[1]
Employed part-time...	☐[2]
Retired	☐[3] *(Go to question 22.)*
Not currently employed.......................................	☐[4] *(Go to question 22.)*

7. Which **one** of the following best describes your **primary** employer? *(Please select one answer.)*

National Cancer Institute (NCI)	☐[1] *(Go to question 10.)*
National Institutes of Health (NIH) other than NCI..	☐[2] *(Go to question 10.)*
Government agency other than NIH	☐[3] *(Go to question 9.)*
University or some other academic institution.....	☐[4] *(Go to question 8.)*
Independent cancer research center or some other health research institution	☐[5] *(Go to question 9.)*
Health care clinic or hospital...............................	☐[6] *(Go to question 9.)*
A foundation or professional association...........	☐[7] *(Go to question 9.)*
Private company...	☐[8] *(Go to question 9.)*
Self-employed	☐[9] *(Go to question 10.)*

8. What is your tenure status? *(Please select one answer.)*

   Tenured .... ☐ 1
   On the tenure track... ☐ 2
   Not on the tenure track.. ☐ 3

9. What is the name of your primary employer? ____

10. What is the title of your current position? ____

11. How long have you been at your **current** job?

   ☐☐year(s) ☐☐ month(s)

12. Are you affiliated with an NCI-designated Cancer Center? (yes/no) *(If no, skip the next two questions)*

13. If yes, which one? ____

14. What is your role or affiliation with the Cancer Center?

   Member.. ☐ 1
   Lead for specialized area (e.g., community engagement) *(Please specify role/title.)* .... ☐ 2
   Cancer Center Senior Leadership *(Please specify role/title.)* ……………………… ☐ 3
   Director .... ☐ 4
   Other *(Please specify.)* .... ☐ 5

15. In what discipline(s) does your current work primarily fall? *(Please select all that apply.)*

   Behavioral or social sciences .... ☐ 1
   Biological or biomedical sciences....................... ☐ 2
   Epidemiology and/or public health .... ☐ 3
   Mathematical sciences .... ☐ 4
   Medicine... ☐ 5
   Nutrition sciences... ☐ 6
   Physical sciences.. ☐ 7
   Other *(Please specify.)* .... ☐ 8

16. For this question, please exclude time spent on cancer treatment or cancer treatment research. **Approximately** what percentage of your current work is done **in cancer prevention and control?** *(Please select one answer.)*

   None .... ☐ 1 *(Go to question 17.)*
   A small percentage (1%–25%) .... ☐ 2 *(Go to question 18.)*
   A moderate percentage (26%–50%) .... ☐ 3 *(Go to question 18.)*
   A large percentage (51%–75%) .... ☐ 4 *(Go to question 18.)*
   A very large percentage (76%–100%) .... ☐ 5 *(Go to question 18.)*

17. What are the reasons you are **not** currently working in the field of cancer prevention or control? *(Please select all that apply and then go to question 18.)*

   A suitable job in the field was not available .... ☐ 1
   A better opportunity outside of the field was available... ☐ 2
   My career or professional interests changed........ ☐ 3
   Perceived exclusion within the scientific community…………………………………… ☐ 4

Lack of funding or other support for cancer prevention work .... ☐5
Other professional or personal reasons *(Please specify.)* .... ☐6

18. **Approximately** what percentage of your time in your current job is spent on **all research and research support activities, not just those in cancer prevention and control**? Please include time spent conducting research yourself, as well as time spent supporting the research of others through activities such as research management, monitoring, reviewing, funding, analysis, dissemination, mentoring, and other research support activities. *(Please select one answer.)*

    None .... ☐1 *(Move to question 20.)*
    A small percentage (1%–25%) .... ☐2
    A moderate percentage (26%–50%) .... ☐3
    A large percentage (51%–75%) .... ☐4
    A very large percentage (76%–100%) .... ☐5

19. **Approximately** what percentage of your time in your current job is spent on **research and research support activities in cancer prevention and control?** Please include time spent conducting research yourself, as well as time spent supporting the research of others through activities such as research management, monitoring, reviewing, funding, analysis, dissemination, mentoring, and other research support activities. *(Please select one answer.)*

    None .... ☐1
    A small percentage (1%–25%) .... ☐2
    A moderate percentage (26%–50%) .... ☐3
    A large percentage (51%–75%) .... ☐4
    A very large percentage (76%–100%) .... ☐5

20. **Approximately** what percentage of your time in your current job is spent on advising students and fellows? *(Please select one answer.)*

None .... [1] ☐
A small percentage (1%–25%) .... [2] ☐
A moderate percentage (26%–50%) .... [3] ☐
A large percentage (51%–75%) .... [4] ☐
A very large percentage (76%–100%) .... [5] ☐

21. **Multidisciplinary** activities are activities that involve several academic disciplines or professional specializations. To what extent do you currently engage in the following collaborative and multidisciplinary activities with other professionals? *(Please select one answer in each row.)*

**Professional activity**	**Not applicable**	**Not at all**	**A small extent**	**A moderate extent**	**A large extent**	**A very large extent**
a. Incorporate research from multiple fields/disciplines in your work	☐ 1	☐ 2	☐ 3	☐ 4	☐ 5	☐ 6
b. Collaborate with professionals from multiple disciplines..................................	☐ 1	☐ 2	☐ 3	☐ 4	☐ 5	☐ 6
c. Manage and/or lead professionals from multiple disciplines..................................	☐ 1	☐ 2	☐ 3	☐ 4	☐ 5	☐ 6
d. Present at multidisciplinary conferences, symposia, workshops or meetings	☐ 1	☐ 2	☐ 3	☐ 4	☐ 5	☐ 6
e. Publish in multidisciplinary journals or publications...	☐ 1	☐ 2	☐ 3	☐ 4	☐ 5	☐ 6
e. Publish with professionals from multiple disciplines……………………………….	☐ 1	☐ 2	☐ 3	☐ 4	☐ 5	☐ 6
f. Lead or contributed to a multidisciplinary training program…………………………	☐ 1	☐ 2	☐ 3	☐ 4	☐ 5	☐ 6

CAREER ACTIVITIES

This section asks more detailed information about the types of activities you engage in as part of your work.

22. To what extent have you had a role in the following professional activities **during your career?** *(Please select one answer in each row.)*

**Professional activity**	**Not applicable**	**Not at all**	**A small extent**	**A moderate extent**	**A large extent**	**A very large extent**
a. Pursued a new theoretical direction or addressed a topic previously unexplored in cancer research	☐ 1	☐ 2	☐ 3	☐ 4	☐ 5	☐ 6
b. Made a significant contribution to a scientific breakthrough in cancer research	☐ 1	☐ 2	☐ 3	☐ 4	☐ 5	☐ 6
c. Made a significant contribution to advancing innovative ideas in cancer research	☐ 1	☐ 2	☐ 3	☐ 4	☐ 5	☐ 6
d. Addressed key knowledge gaps in cancer research	☐ 1	☐ 2	☐ 3	☐ 4	☐ 5	☐ 6
e. Developed funding initiatives to address knowledge gaps in cancer research	☐ 1	☐ 2	☐ 3	☐ 4	☐ 5	☐ 6

23. For this question, please answer only for activities that occurred after completing your doctoral degree and postdoctoral work, if applicable. **During the past five years,** how many times have you engaged in the following publication activities? *(Please select one answer on each row.)*

**Publication activity**	**Not applicable**	**None**	**Once**	**Two or three times**	**Four or five times**	**Six or more times**
a. Authored or co-authored a paper in a published peer-reviewed journal.....................	☐ 1	☐ 2	☐ 3	☐ 4	☐ 5	☐ 6
b. Authored or co-authored a chapter in a published book...	☐ 1	☐ 2	☐ 3	☐ 4	☐ 5	☐ 6
c. Authored or co-authored a published book....	☐ 1	☐ 2	☐ 3	☐ 4	☐ 5	☐ 6
d. Authored or co-authored a technical report or white paper	☐ 1	☐ 2	☐ 3	☐ 4	☐ 5	☐ 6
e. Authored or co-authored a published presentation abstract or report……………….	☐ 1	☐ 2	☐ 3	☐ 4	☐ 5	☐ 6
f. Authored or co-authored a peer-reviewed pre-print paper……………………………………	☐ 1	☐ 2	☐ 3	☐ 4	☐ 5	☐ 6

24. For this question, please answer only for activities that occurred after completing your doctoral degree and postdoctoral work and exclude presentations given by your students, postdocs and/or staff, if applicable. **During the past five years,** how many times have you personally engaged in the following presentation activities? *(Please select one answer on each row.)*

**Presentation activity**	**Not applicable**	**None**	**Once**	**Two or three times**	**Four or five times**	**Six or more times**
a. Presented at a professional conference or scientific meeting	☐1	☐2	☐3	☐4	☐5	☐6
b. Chaired a session or workshop at a professional conference or scientific meeting	☐1	☐2	☐3	☐4	☐5	☐6
c. Organized a session or workshop at a professional conference or scientific meeting	☐1	☐2	☐3	☐4	☐5	☐6
d. Organized a professional conference or scientific meeting..	☐1	☐2	☐3	☐4	☐5	☐6

25. For this question, please answer only for activities that occurred after completing your doctoral degree and postdoctoral work, if applicable. **During the past five years,** how many times have you engaged in the following community service activities? *(Please select one answer on each row.)*

**Community service activity**	**Not applicable**	**None**	**Once**	**Two or three times**	**Four or five times**	**Six or more times**
a. Advised or presented information to a patient advocacy or support group..................	☐1	☐2	☐3	☐4	☐5	☐6
b. Translated cancer research information for a lay audience…...	☐1	☐2	☐3	☐4	☐5	☐6
c. Served on a local health advisory board, panel, or committee	☐1	☐2	☐3	☐4	☐5	☐6
d. Served on a national health advisory board, panel, or committee	☐1	☐2	☐3	☐4	☐5	☐6

26. For this question, please answer only for activities that occurred after completing your doctoral degree and postdoctoral work, if applicable. During **the past five years,** how many times have you engaged in the following other professional activities? *(Please select one answer on each row.)*

Other professional activity	Not applicable	None	Once	Two or three times	Four or five times	Six or more times
a. Established or appointed to a working group on cancer research	☐1	☐2	☐3	☐4	☐5	☐6
b. Served as a reviewer for a journal	☐1	☐2	☐3	☐4	☐5	☐6
c. Served as an editor of a journal or served on a journal review board	☐1	☐2	☐3	☐4	☐5	☐6
d. Led or co-led a clinical trial	☐1	☐2	☐3	☐4	☐5	☐6
e. Received a competitive grant, contract, or subcontract for your work	☐1	☐2	☐3	☐4	☐5	☐6
f. Filed or received a patent	☐1	☐2	☐3	☐4	☐5	☐6
g. Developed a prototype, technology, or marketable product	☐1	☐2	☐3	☐4	☐5	☐6
h. Served as editor of a training manual or textbook	☐1	☐2	☐3	☐4	☐5	☐6
i. Other professional activity *(Please specify.)*	☐1	☐2	☐3	☐4	☐5	☐6

27. What is your current annual salary, including any bonuses you have received? *(Please select one answer.)*

- Less than $50,000 ☐1
- $50,000–$74,999 ☐2
- $75,000–$99,999 ☐3
- $100,000–$124,999 ☐4
- $125,000–$149,999 ☐5
- $150,000–$174,999 ☐6
- $175,000–$199,999 ☐7
- $200,000–$224,999 ☐8
- $225,000 or more ☐9
- Not applicable ☐10

28. Have you ever moved institutions for professional advancement reasons?

- Yes ☐1
- No ☐2

29. Do you feel that your salary is generally competitive with others in similar positions?

- Yes ☐1
- No ☐2
- Do not know ☐3

30. Have the following occurred in your career since completing your doctoral degree and postdoctoral work, if applicable? *(Please select one answer on each row.)*

	Yes	No
Advanced to a more senior-level position...........	☐ 1	☐ 2
Assumed a role as a project leader (e.g., technical group leader)....................................	☐ 1	☐ 2
Assumed leadership or management responsibilities (e.g., section chief)................	☐ 1	☐ 2
Served as a mentor to others in your organization...	☐ 1	☐ 2
Assumed other leadership roles (e.g., led a committee)	☐ 1	☐ 2

31. How satisfied are you with the progression of your career to this point? *(Please select one answer.)*

Not at all.. ☐ 1
A little satisfied .... ☐ 2
Somewhat satisfied... ☐ 3
Very satisfied .... ☐ 4
Extremely satisfied... ☐ 5

PROFESSIONAL ASSOCIATIONS AND AWARDS

This section asks about your participation in professional associations and awards you may have received for your work.

32. How many professional associations (e.g., American Association for Cancer Research, American Medical Association) are you **currently** a member of? *(Please select one answer.)*

None .... ☐ 1 *(Go to question 35)*
One .... ☐ 2
Two .... ☐ 3
Three .... ☐ 4
Four or more.. ☐ 5

33. Of the professional associations listed previously, how many are focused on cancer research? __________

34. For this question, please answer only for activities that occurred after completing your doctoral degree and postdoctoral work, if applicable. **During the past five years,** have you held either a volunteer or elected leadership position in a professional association? *(Please select one answer on each row.)*

	Yes	No
A volunteer leadership position	☐ 1	☐ 2
An elected leadership position	☐ 1	☐ 2

35. For this question, please answer only for activities that occurred after completing your doctoral degree and postdoctoral work, if applicable. **During the past five years,** have you received a professional award related to your work?

Yes *(Please specify.)* .... ☐ 1
No .... ☐ 2

____

PROGRAM BENEFITS *(Display for Alumni group only; Applicant group go to question 41)*

This section asks questions about benefits you received from participating in the Cancer Prevention Fellowship Program.

36. **While a fellow,** how beneficial was the Cancer Prevention Fellowship Program to your knowledge, skills, and research in the following areas? *(Please select one answer on each row.)*

Area	Don't remember	Not at all beneficial	A little beneficial	Somewhat beneficial	Very beneficial	Extremely beneficial
a. Scientific subject matter knowledge/expertise............................	☐ 1	☐ 2	☐ 3	☐ 4	☐ 5	☐ 6
b. Scientific identity in the cancer prevention research field …………….	☐ 1	☐ 2	☐ 3	☐ 4	☐ 5	☐ 6
c. Knowledge/expertise in public health...	☐ 1	☐ 2	☐ 3	☐ 4	☐ 5	☐ 6
d. Research skills and/or techniques..........	☐ 1	☐ 2	☐ 3	☐ 4	☐ 5	☐ 6
e. Experience using specialized equipment and/or technology	☐ 1	☐ 2	☐ 3	☐ 4	☐ 5	☐ 6
f. Confidence in performing research	☐ 1	☐ 2	☐ 3	☐ 4	☐ 5	☐ 6
g. The overall quality of your research......	☐ 1	☐ 2	☐ 3	☐ 4	☐ 5	☐ 6
h. The specific direction of your research...	☐ 1	☐ 2	☐ 3	☐ 4	☐ 5	☐ 6
i. The progress of your research	☐ 1	☐ 2	☐ 3	☐ 4	☐ 5	☐ 6
j. Your ability to conduct independent research	☐ 1	☐ 2	☐ 3	☐ 4	☐ 5	☐ 6
k. Contacts who advised or collaborated with you on your research	☐ 1	☐ 2	☐ 3	☐ 4	☐ 5	☐ 6
l. Research collaboration skills………….	☐ 1	☐ 2	☐ 3	☐ 4	☐ 5	☐ 6

37. **While a fellow,** how beneficial was the Cancer Prevention Fellowship Program in the following areas pertaining to other professional-related knowledge and skills? *(Please select one answer on each row.)*

Skill	Don't remember	Not at all beneficial	A little beneficial	Somewhat beneficial	Very beneficial	Extremely beneficial
a. Publication skills	☐ 1	☐ 2	☐ 3	☐ 4	☐ 5	☐ 6
b. Presentation skills.................................	☐ 1	☐ 2	☐ 3	☐ 4	☐ 5	☐ 6
c. Grant and/or contract writing skills.......	☐ 1	☐ 2	☐ 3	☐ 4	☐ 5	☐ 6
d. Mentoring skills	☐ 1	☐ 2	☐ 3	☐ 4	☐ 5	☐ 6
e. Leadership and/or management skills ...	☐ 1	☐ 2	☐ 3	☐ 4	☐ 5	☐ 6
f. Professional networking skills…………	☐ 1	☐ 2	☐ 3	☐ 4	☐ 5	☐ 6

38. How beneficial was the Cancer Prevention Fellowship Program to you in terms of providing the following career-related benefits? *(Please select one answer on each row.)*

Benefit	Not at all beneficial	A little beneficial	Somewhat beneficial	Very beneficial	Extremely beneficial	Not applicable
a. Securing your first position after the fellowship	☐ 1	☐ 2	☐ 3	☐ 4	☐ 5	☐ 6
b. Securing subsequent positions following your first position after the fellowship	☐ 1	☐ 2	☐ 3	☐ 4	☐ 5	☐ 6
c. Providing you with contacts that have helped you find employment	☐ 1	☐ 2	☐ 3	☐ 4	☐ 5	☐ 6
d. Positively influencing your ability to obtain funding for your work...............................	☐ 1	☐ 2	☐ 3	☐ 4	☐ 5	☐ 6
e. Helping you achieve your career goals	☐ 1	☐ 2	☐ 3	☐ 4	☐ 5	☐ 6
f. Influencing the specific direction of your current research..................................	☐ 1	☐ 2	☐ 3	☐ 4	☐ 5	☐ 6
g. Influencing the progress of your current research	☐ 1	☐ 2	☐ 3	☐ 4	☐ 5	☐ 6
h. Influencing your career trajectory	☐ 1	☐ 2	☐ 3	☐ 4	☐ 5	☐ 6

39. Looking back on your career thus far, would you make the same decision to participate in the Cancer Prevention Fellowship Program?

Yes.. ☐ 1
No .... ☐ 2

40. Have you ever encouraged someone else to apply for the Cancer Prevention Fellowship Program?

Yes.. ☐ 1
No .... ☐ 2

REFLECTIONS AND RECOMMENDATIONS *(Display only first question of this section to Applicant group; Alumni group will receive all questions and introduction language.)*

This section asks a few open-ended questions about significant accomplishments in your career, your opinion about the most valuable aspect of the program, and your recommendations for program improvements.

41. What do you consider to be the two or three most important accomplishments in your career?

(Go to Demographics section if Applicant group)

42. What was the single most valuable aspect of the Cancer Prevention Fellowship Program to you?

43. What is the single most important improvement you would like made in the Cancer Prevention Fellowship Program?

44. If you could make other improvements to the Cancer Prevention Fellowship Program, what would they be?

DEMOGRAPHICS

The last section of the survey asks about demographic information, including your education.

45. What terms best express how you describe your sex? *(Please select all that apply.)*

Male .... ☐ 1
Female .... ☐ 2
Prefer not to answer .... ☐ 3

46. Which categories describe you? Select all that apply. (*You may select more than one category.)*

American Indian or Alaska Native……….............. ☐ 1
Hispanic, Latino, or Spanish.................................. ☐ 2
Middle Eastern or North African …...................... ☐ 3
Asian .... ☐ 4
Black, African American, or African.................... ☐ 5
Native Hawaiian or Other Pacific Islander…......... ☐ 6
White... ☐ 7
None of these fully describe me *(Please specify.)* …..... ☐ 8
Prefer not to answer ………………………………… ☐ 9

47. What is your birth year? [Drop down with years]

48. Which of the following doctoral degrees have you received and in what year did you receive them? *(Please select all that apply.)*

		**Year of most recent degree**
PhD	☐ 1	______
ScD	☐ 2	______
MD	☐ 3	______
DO	☐ 4	______
DrPH	☐ 5	______
DDS	☐ 6	______
DMD	☐ 7	______
JD	☐ 8	______
DVM	☐ 9	______
Other (*Please specify.*)	☐ 10	______

49. Which of the following other degrees or certifications do you have?

- MS … ☐ 1
- MA ☐ 2
- MPH ☐ 3 *(If in alumni group, go to question 50)*
- MBA ☐ 4
- NP ☐ 5
- RD ☐ 6
- RN ☐ 7
- Other (*Please specify.)* ______ ☐ 8

50. Did you earn your MPH through the CPFP program?

- Yes ☐ 1
- No ☐ 2

51. What is your current marital status?

- Married ☐ 1
- Divorced ☐ 2
- Separated ☐ 3
- Never married ☐ 4
- Living with partner ☐ 5
- Prefer not to answer ☐ 6

52. Not including yourself, how many other people live at home with you?

☐☐

53. Not including yourself, how many other people under the age of 18 years old live at home with you?
